# Distribution of cone density, spacing and arrangement in adult healthy retinas with adaptive optics flood illumination

**DOI:** 10.1371/journal.pone.0191141

**Published:** 2018-01-16

**Authors:** Richard Legras, Alain Gaudric, Kelly Woog

**Affiliations:** 1 Laboratoire Aimé Cotton, CNRS, Université Paris-Sud, ENS Paris-Saclay, Université Paris-Saclay, Orsay, France; 2 Université Paris Diderot - APHP Hôpital Lariboisière, Paris, France; Instituto Murciano de Investigacion y Desarrollo Agrario y Alimentario, SPAIN

## Abstract

The aim of this article is to analyse cone density, spacing and arrangement using an adaptive optics flood illumination retina camera (rtx1^™^) on a healthy population. Cone density, cone spacing and packing arrangements were measured on the right retinas of 109 subjects at 2°, 3°, 4°, 5° and 6° of eccentricity along 4 meridians. The effects of eccentricity, meridian, axial length, spherical equivalent, gender and age were evaluated. Cone density decreased on average from 28 884 ± 3 692 cones/mm^2^, at 2° of eccentricity, to 15 843 ± 1 598 cones/mm^2^ at 6°. A strong inter-individual variation, especially at 2°, was observed. No important difference of cone density was observed between the nasal and temporal meridians or between the superior and inferior meridians. However, the horizontal and vertical meridians differed by around 14% (T-test, p<0.0001). Cone density, expressed in units of area, decreased as a function of axial length (r^2^ = 0.60), but remained constant (r^2^ = 0.05) when cone density is expressed in terms of visual angle supporting the hypothesis that the retina is stretched during the elongation of the eyeball. Gender did not modify the cone distribution. Cone density was slightly modified by age but only at 2°. The older group showed a smaller density (7%). Cone spacing increased from 6,49 ± 0,42 μm to 8,72 ± 0,45 μm respectively between 2° and 6° of eccentricity. The mosaic of the retina is mainly triangularly arranged (i.e. cells with 5 to 7 neighbors) from 2° to 6°. Around half of the cells had 6 neighbors.

## Introduction

The retinal structure of the human eye has been extensively studied. First measurements came from histology where post mortem retinal data were analyzed [1, 2]. Naturally, these studies concerned few subjects, always less than 10.

In-vivo measurements permit to obtain data on large populations. The two main devices used to acquire in-vivo images of the retina on a large population are the Adaptive Optics Scanning Laser Ophthalmology (AOSLO) [3–10] and the adaptive optics flood-illumination retinal cameras (e.g. rtx1^™^ device) [11–22]. Both systems used adaptive optics to improve the quality of the images.

Up to now, the biggest database was published by Park et al. in 2013 [8]. They used an AOSLO system to measure and compare in-vivo cone density with many parameters such as retinal eccentricity, age, axial length (AL), refractive error, gender, race/ethnicity and ocular dominance on 192 subjects.

Even if the main characteristics of the retina are known, some discrepancies between studies leave questions unanswered.

As an example, whereas Park et al.[8] did not measure an important difference in cone density between the horizontal and the vertical meridians (i.e. around 2% with AOSLO, from 1.7° to 5°), Curcio et al. [2] (histology, from 0° to 7°), Dabir et al. [21] (rtx1^™^, between 2° to 3°), Chui et al. [5] (AOSLO, from 3° to 6°), Feng et al. [20] (rtx1^™^, from 2° to 5.5°), Song et al., [7] (AOSLO, from 0.6° to 7°), and Dabir et al. [15] (rtx1^™^, from 2° to 3°) measured a significant difference of respectively 9%, 9.3%, 11%, 11%, 12.5% and 13%.

Another example is the role of the axial length (AL) on cone density expressed in metric units that should be clarified. Indeed, some authors found an important correlation (r^2^ = 0.56 at 3°, 0.37 at 5° and 0.23 at 7° [5]; r^2^ = 0.40 at 2° [23]; r^2^ = 0.75 at 1° [6]) while others found low correlation (r^2^ = 0.14 at ~2°, 0.022 at 3° and 0.037 at 5° [8]; r^2^ = 0.16 at 2° and 0.14 at 3° [19]).

Another uncertainty concerns the effect of age on the cone density. Song et al. [7], who measured cone density between 0.6° and 7.4°, observed a statistically significant difference of cone density up to ~ 1.5° between younger (22–35 years) and older (50–65 years) subjects. Panda-Jonas et al. [24] measured cone density (histology) between ~ 7° and ~ 69°. They found a statistically significant difference from 5 mm of eccentricity (~ 17°) to 17 mm (~ 58°). Other authors [2, 8, 22] did not measure any significant difference with age.

Moreover, the majority of vision loss in developed countries is caused by retinal diseases that affect retinal photoreceptors, including age-related macular degeneration (AMD) and diabetic retinopathy (DR). Measurement of cone density in the fovea and parafoveal region is one of the parameter which could be used in the future to give us direct information for detection of retinal pathologies and to assess the progression of retinal atrophy. As a consequence, it is useful as now to establish the normal distribution of cones in the macular area using a clinically easy to use device of AO fundus camera.

The aim of this study was to measure cone photoreceptor characteristics in a large healthy adult population, with a flood illuminated adaptive optics camera (rtx1^™^, Imagine Eyes, Orsay, France). The rtx1^™^ device is a commercial system which is able to image the microscopic structure of the retina. The main advantage of this device is its ability to be used in any clinical practice. The effects of eccentricity, meridians, axial length, gender and age on cone density, cone spacing and packing arrangements were evaluated along the horizontal and vertical meridians.

## Materials and methods

### Subjects

We recruited 117 subjects on a voluntary basis. Eight of them were excluded due to the presence of an abnormal retina (see the exclusion criteria). We finally tested 109 healthy subjects (71 men and 38 women), aged from 20 to 59 years old (mean 38.4 years; SD 11.7 years), divided into 4 age groups (28 subjects in group 1 [18; 30 years[; 28 subjects in group 2 [31; 40 years[; 28 subjects in group 3 of the [41; 50 years [and 25 subjects in group 4 of the [51; 60 years]).

The experimental protocol was approved by the ethics committee EA 4532 of the University Paris-Sud. The tenets of the Declaration of Helsinki were followed. After receiving a verbal and written explanation of the nature and possible consequences of the study, all subjects provided written informed consent.

All subjects received a complete eye examination including a fundus and slit-lamp examination.

Based on digital non-mydriatic fundus photography, subjects with retinal pathology such as macular degeneration, glaucoma, diabetic or hypertensive retinopathy were excluded. Subjects showing binocular abnormalities such as strabismus or nystagmus were also excluded. In addition, subjects should have clear ocular media.

Their best corrected visual acuity (Freiburg Visual Acuity Test) should be 0.00 logMAR or better (mean -0.12 logMAR; SD 0.06 logMAR; min 0.00 logMAR; max -0.31 logMAR). Their subjective refractive error, should be between -6D and +4D, and astigmatism could not exceed 3D. Axial length was measured with the IOLmaster (Carl Zeiss Meditec). The axial length ranged from 22 to 26 mm (mean 23.95 mm; SD 0.83 mm).

The images were acquired on the right eye of each subject. The pupil size measured under the conditions of experiment should be larger than 4.5 mm to improve the quality of the images. The clinical characteristics of the subjects are detailed in Table 1.

**Table 1:** Details of the demographic population.

Parameters	Values
**Number of subject (eye)**	109 (109)
**Sex (M/F)**	n = 71 (65%) / n = 38 (35%)
**Age (mean ± SD)**	38.4 ± 11.7
**Group 1 [18; 30 years [(mean ± SD)**	n = 28 (22.68 ± 2.39)
**Group 2 [31; 40 years [(mean ± SD)**	n = 28 (34.54 ± 2.91)
**Group 3 [41; 50 years [(mean ± SD)**	n = 28 (44.21 ± 1.95)
**Group 4 [51; 60 years] (mean ± SD)**	n = 25 (53.64 ± 2.55)
**Axial length in mm (mean ± SD; range)**	23.95 ± 0.83; 22.01–26.00
**Keratometry in mm (mean ± SD; range)**	7.83 ± 0.27; 7.23–8.81
**Spherical equivalent in Diopter (mean ± SD; range)**	-0.6 ± 1.7; -5.50–+3.50
**Visual acuity in logMAR (mean ± SD; range)**	-0.12 ± 0.06; 0.00–-0.31

M = male; F = female; SD = standard deviation.

### Retinal imaging with the flood illuminated AO retinal camera (rtx1^™^)

We acquired on each eye a series of images of the retina using a commercially available flood-illuminated AO retinal camera (rtx1^™^, Imagine Eyes, Orsay, France).

For one final image, a set of 40 raw images of the same retinal area were acquired at a rate of 9.5 frames per second with an exposure time of 10 ms. The final AO image was averaged in a 4 x 4 degrees field (i.e. 1500 x 1500 pixels).

Retinal images of the right eye were acquired at 0°, 2°, 4° and 6° of eccentricity, along the 4 meridians (nasal, temporal, superior and inferior). The subject had to fixate a yellow cross controlled by the operator. Subject’s defocus term was compensated by a Badal setup.

The rtx1^™^ is based on a flood illumination design using the principle of reflectance imaged in the near-infrared (850 nm). Raw images are acquired by a low noise CCD camera. The high resolution of this instrument is obtained by an Adaptive Optics arrangement which is based on a Shack-Hartmann wavefront sensor (Haso3-32, Imagine Optic, France) illuminated at 780 nm and a deformable mirror (mirao52e, Imagine Eyes, France). A full description of the rtx1^™^ has been previously published [25].

Cones are automatically detected by a software provided by the manufacturer (AOdetect Mosaic b13, Imagine Eyes, France). The background of the image is removed and the histogram is stretched, then adaptive [26] and multiple—scale [27] digital filters are applied to the resulting image. The local maxima of the resulting filtered image were detected. Their spatial distribution was analyzed in terms of inter-cones spacing, local cell density and number of nearest neighbors using Delaunay triangulation [28] and Voronoi diagrams [6].

Cone mosaic metrics (local density, spacing and number of neighbours) were analysed at each degree from 2° to 6° of eccentricity.

**Fig 1:**
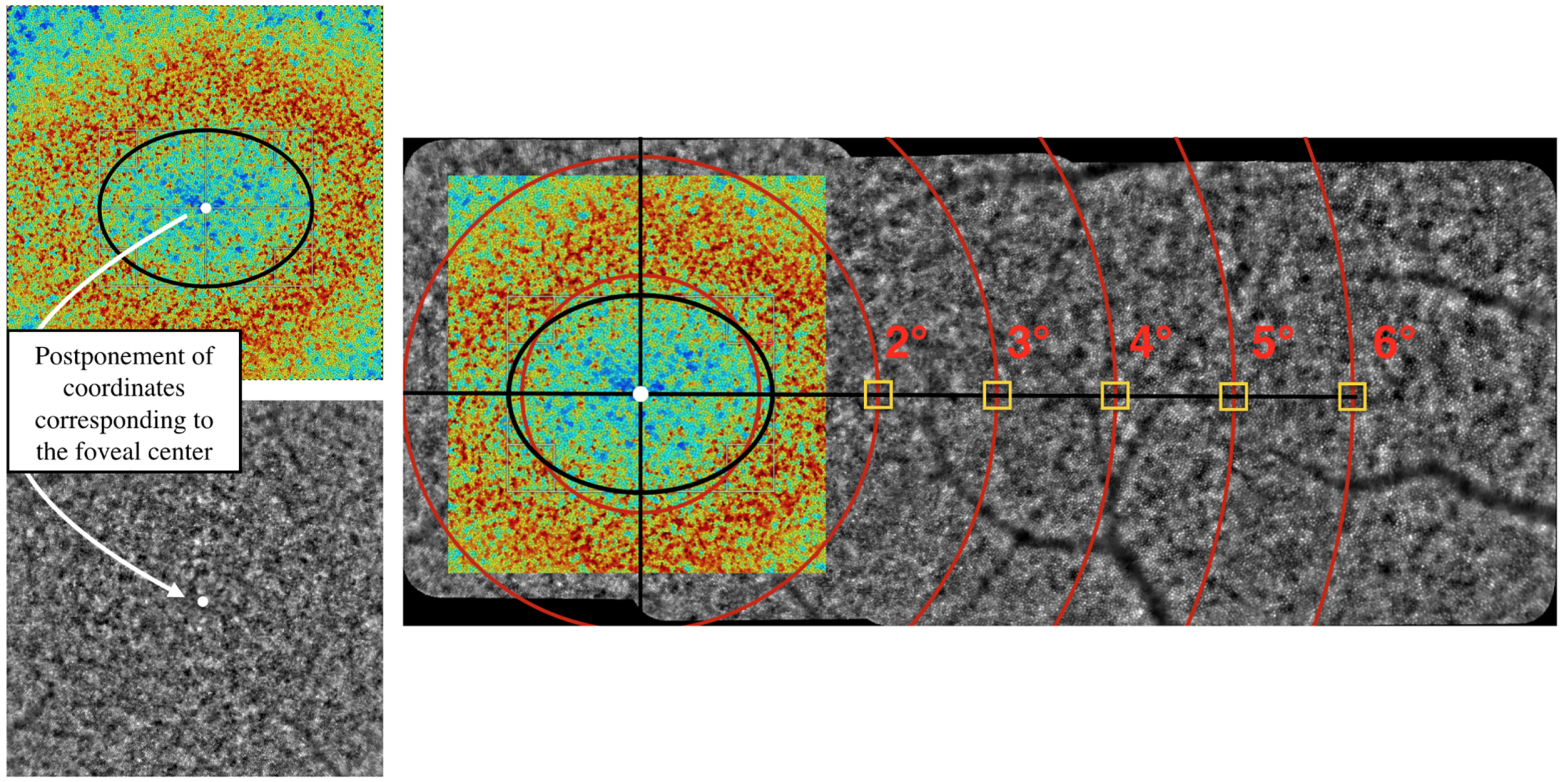
Illustration of the method used to determine the foveal center. The montage allows to accurately determine the eccentricities. The Region Of Interest (ROI) of 80 x 80 pixels is represented by a yellow square.

It is known that the isodensity contours of cone photoreceptors is elongated along the horizontal axis [2]. Thus, we defined the center of the fovea (i.e. coordinate (0;0)) as the center of the ellipse surrounding the maximum cone density, highlighted with a Voronoï density analysis (Fig 1). Indeed, cones within the 2° central area (i.e. up to 1° from the center of the fovea), cannot be resolved due to the limit of resolution of the device. This appeared on the image as a blue/green central part (i.e. the cones were not resolved involving a false low density) surrounding by a red/orange elliptical area corresponding to high cone densities.

Then, a montage of the 4°x4° final AO images was done using the I2K retina^®^ software (Dual Align). Eccentricities from the foveal center, as previously defined, were accurately determined on the montage. We favored to measure the cone parameters on a montage of images to avoid any important errors of fixation due to eye movements.

Cone density, cone spacing and cone arrangement were analyzed with AOdetect in a Region Of Interest (ROI) of 80 x 80 pixels, corresponding to 62 x 62 μm on the retina for an axial length of 23.80 mm (Yellow square in the Fig 1). When the ROI fell on a shadow or a black area, it was slightly shifted towards a measurable area. Following this automatic detection, a manual correction of cone counts was performed by an experiment investigator at each retinal eccentricity and in all meridians.

To evaluate the repeatability of the rtx1^™^ device, we performed a one week test-retest on 10 subjects. Images of the temporal retina were acquired at 2°, 4° and 6° of eccentricity and cones density were calculated. The difference of the two measurements averaged among the eccentricities was less than 6% confirming the good repeatability of the rtx1^™^ measured by Bidaut Garnier et al., in 2014 [29].

We also tested the reproducibility of the method. Three investigators, including two inexperienced investigators, manually corrected the automatic detection of cones on 30 images acquired on 10 subjects at 2°, 4° and 6° along the temporal meridian. Placement of the ROI in the case of shadow or a black area and manual correction were performed by the investigators. We observed an averaged difference of less than 4% between investigators, ranging from 4.6% at 2° to 2.5% at 6°.

## Results

Averaged on the four meridians and the five eccentricities, the mean difference between the automatic and manual corrected detection was 6% (i.e. under-estimation by the automatic detection). A larger discrepancy (i.e. around 10%) was obtained at 2°. The following results take into account the manual correction.

**Fig 2:**
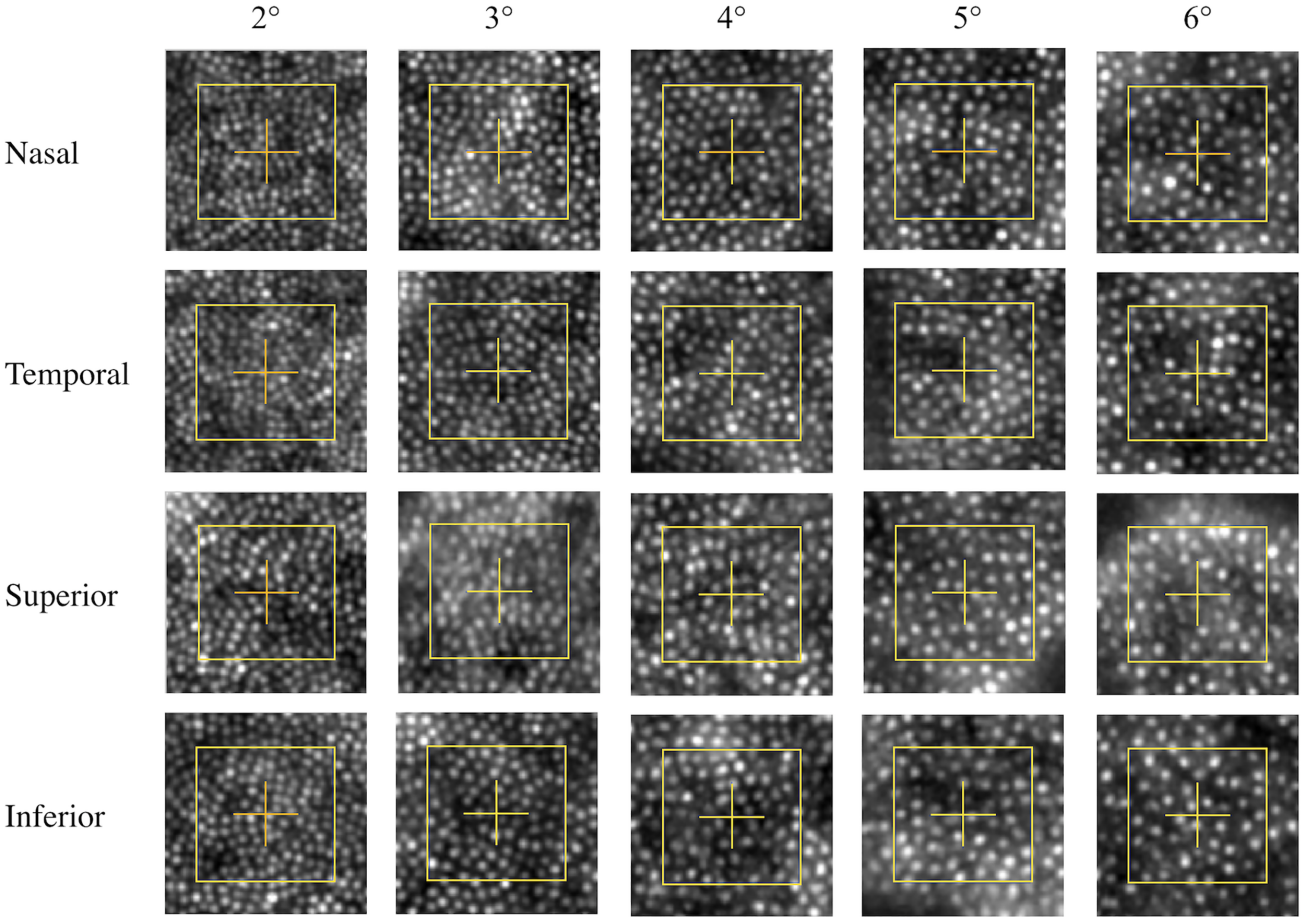
Images of ROI (yellow square) analysed at 2°, 3°, 4°, 5° and 6° (columns) along the nasal, temporal, superior and inferior meridians of one typical subject. We can observed a decrease of cone density with eccentricity as well as a difference of cone density between the horizontal and vertical meridians.

Table 2 shows the mean, standard deviation, minimum and maximum cone density measured at all eccentricities and meridians. Fig 2 represents images of the ROI analysed at 2°, 3°, 4°, 5° and 6° (columns) along the nasal, temporal, superior and inferior meridians of one typical subject.

**Table 2:** Mean ± standard deviation (minimum—maximum) cone density including manual correction for all eccentricities and all meridians, expressed in cone/mm^2^ and cone/deg^2^.

Density		2°	3°	4°	5°	6°	Average
**Nasal** cone/mm^2^		30774 ± 4002 (21379–41244)	23945 ± 2650 (16698–31629)	20046 ± 1989 (15659–25792)	17999 ± 1510 (13735–22566)	16748 ± 1534 (13692–20809)	21890 ± 1997 (16859–28139)
**Temporal** cone/mm^2^		31438 ± 4362 (19254–43391)	24747 ± 2626 (18591–30206)	20072 ± 2033 (14784–25360)	17688 ± 1402 (13751–20382)	16217 ± 1651 (12733–20489)	22050 ± 2071 (17104–26630)
**Superior** cone/mm^2^		26113 ± 3047 (19174–33566)	19558 ± 2045 (13779–24133)	16944 ± 1435 (13164–19994)	15880 ± 1514 (12661–18937)	15394 ± 1622 (11481–19334)	18818 ± 1581 (14532–22138)
**Inferior** cone/mm^2^		27212 ± 3357 (19232–39135)	19511 ± 2088 (14707–26743)	16721 ± 1436 (13101–19647)	15789 ± 1544 (11854–19340)	15014 ± 1585 (11767–18965)	18861 ± 1659 (15092–24512)
**T-test**	**N-T**	p = 0.05	p = 0.001	p = 0.90	p = 0.02	p = 0.001	p = 0.277
	**S-I**	p<0.001	p = 0.88	p = 0.08	p = 0.49	p = 0.012	p = 0.642
	**H-V**	p<0.001	p<0.001	p<0.001	p<0.001	p<0.001	p<0.001
**Nasal** cone/deg^2^		2595 ± 280 (1738–3329)	2021 ± 182 (1607–2519)	1693 ± 147 (1431–2239)	1519 ± 118 (1253–1791)	1411 ± 101 (1175–1650)	1848 ± 131 (1503–2161)
**Temporal** cone/deg^2^		2647 ± 269 (1849–3206)	2089 ± 184 (1735–2592)	1696 ± 157 (1393–2172)	1494 ± 107 (1243–1958)	1369 ±120 (1134–1708)	1861 ± 127 (1615–2160)
**Superior** cone/deg^2^		2205 ± 229 (1596–2910)	1650 ± 139 (1244–1967)	1431 ± 102 (1197–1786)	1342 ± 110 (1123–1680)	1298 ± 103 (1010–1610)	1589 ± 100 (1363–1925)
**Inferior** cone/deg^2^		2294 ± 218 (1792–3003)	1647 ± 140 (1421–2272)	1413 ± 110 (1177–1727)	1333 ± 102 (1065–1539)	1267 ± 99 (994–1521)	1592 ± 90 (1356–1881)
**T-test**	**N-T**	p = 0.07	p = 0.001	p = 0.85	p = 0.01	p = 0.001	p = 0.31
	**S-I**	p<0.001	p = 0.87	p = 0.09	p = 0.45	p = 0.012	p = 0.74
	**H-V**	p<0.001	p<0.001	p<0.001	p<0.001	p<0.001	p<0.001

N = nasal; T = temporal; S = superior; I = inferior; H = horizontal and V = vertical.

We did not observe a difference between the nasal and temporal meridians (T-test, p = 0.28) or between the superior and inferior meridians (T-test, p = 0.64). However, cone densities measured along the horizontal meridian were higher than along the vertical meridian. They differed from 8% at 6° to 25% at 3°. These differences are always highly statistically significant (see Table 2). The coefficient of variation (i.e. Standard Deviation/mean) decreases with eccentricity, from 13% at 2° to 10% at 6° of eccentricity.

When expressed in number of cones per millimeter square, the cone density is well correlated (r^2^ = 0.60) with axial length. When expressed in number of cones per degree square, we did not find any correlation (r^2^ = 0.05). These results are similar for each eccentricity (see Table 3).

**Table 3:** Main effects ANOVA test for age, axial length, gender and spherical equivalent, at each eccentricity. Results and correlations are expressed for metric and visual units.

Main effects ANOVA			2°	3°	4°	5°	6°	All
**Age**	Cone/deg^2^	p	**0.03**	0.91	0.92	0.35	0.52	
		r^2^	0.10	0.01	0.001	0.02	0.01	0.04
	Cone/mm^2^	p	**0.02**	0.91	0.94	0.23	0.62	
		r^2^	0.07	0.01	0.006	0.02	0.01	0.03
**AL**	Cone/deg^2^	p	0.29	0.20	0.32	0.08	0.57	
		r^2^	0	0.03	0.12	0.15	0.04	0.05
	Cone/mm^2^	p	**0.001**	**<0.001**	**<0.001**	**<0.001**	**<0.001**	
		r^2^	0.45	0.51	0.50	0.54	0.60	0.60
**Gender**	Cone/deg^2^	p	0.84	0.40	0.69	0.13	0.33	
	Cone/mm^2^	p	0.83	0.41	0.66	0.09	0.37	
**SE**	Cone/deg^2^	p	**0.046**	0.46	0.18	0.11	0.53	
		r^2^	0.04	0.05	0.12	0.09	0.03	0.09
	Cone/mm^2^	p	0.08	0.56	0.30	0.21	0.63	
		r^2^	0.07	0.11	0.10	0.15	0.18	0.13

AL: Axial Length; SE: Spherical equivalent.

**Fig 3:**
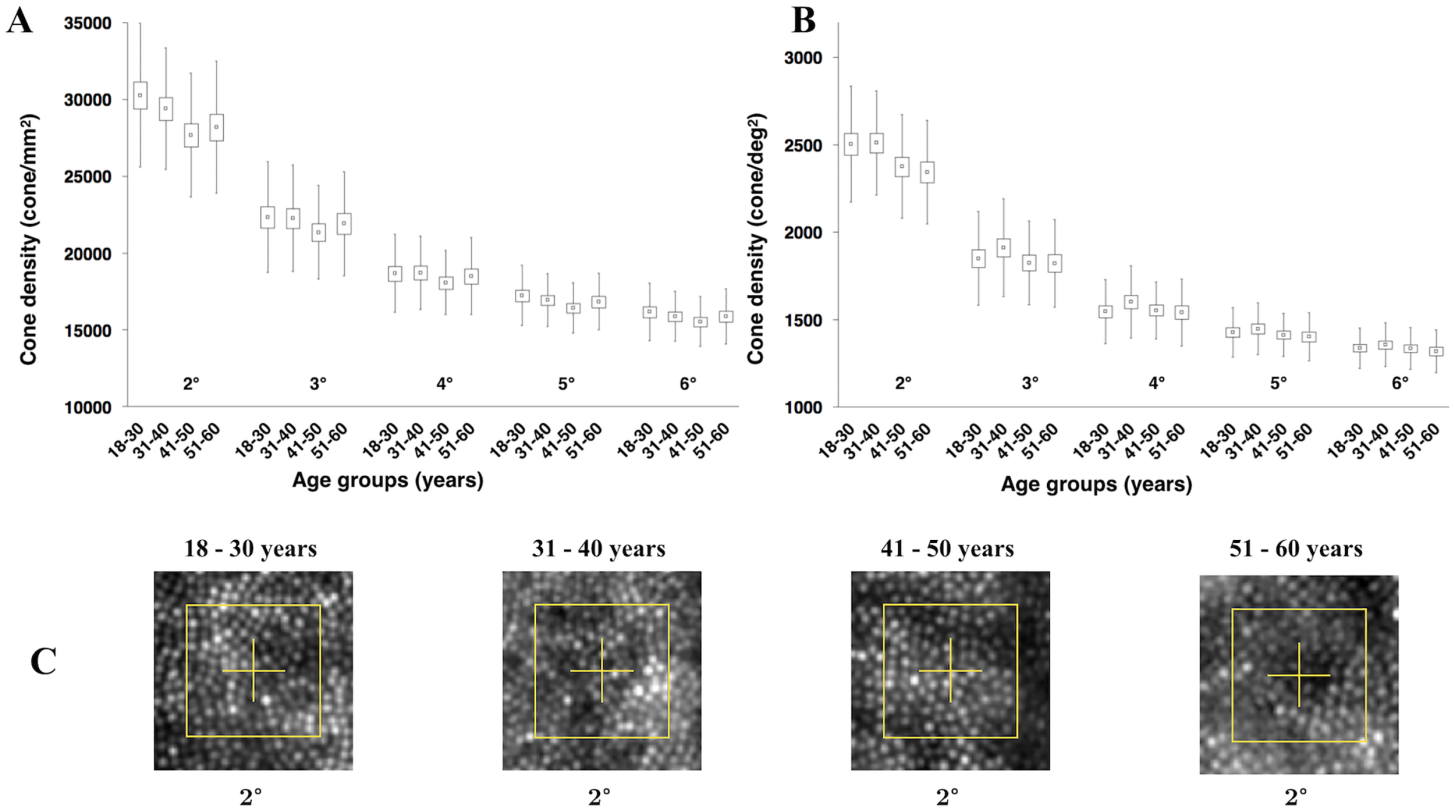
Cone density as a function of age and eccentricity. (A) Results expressed in metric units and (B) in visual units. (C) **Images of ROI (yellow square) analysed at 2° of eccentricity for one subject representative of each age group. Yellow squares represent area of the retina when expressed in visual unit (i.e., 80 pixels or around 13 minutes of arc). We can observe a difference of cone density between the two first groups (i.e., between 18 and 40 years old) and the two last groups (i.e., between 41 and 60 years old).**

Fig 3 shows cone density as a function of age and eccentricity and images of ROI analysed at 2° of eccentricity for one subject representative of each age group. The population was divided into 4 age groups. The main difference between age groups occurred at 2° when expressed either in metric or visual units. Younger subjects show a higher density. No difference was observed at 3° to 6° of eccentricity.

We performed a main effects ANOVA analysis including the age, AL, gender and spherical equivalent (SE) factors. Only age at 2° of eccentricity in both units, SE at 2° in visual unit and AL in metric unit at all eccentricities were statistically significant. Results and correlations are represented in Table 3.

**Fig 4:**
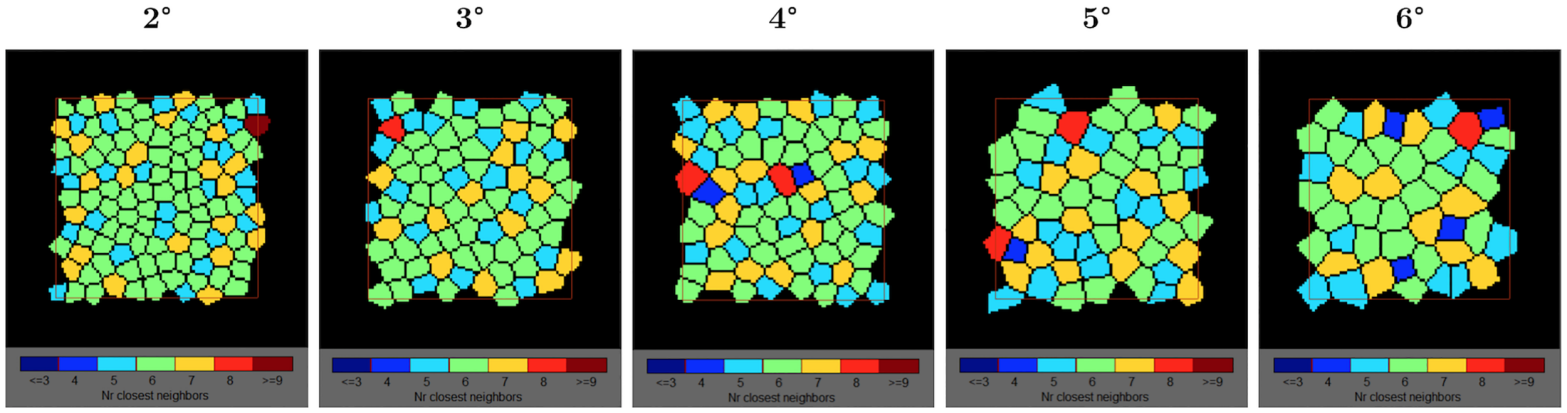
Voronoï analysis with the representation of the spatial distribution of cones. We can observe a small decrease of the proportion of cones with exactly 6 neighbors as a function of eccentricity. In green, cells with 6 neighbors. In orange, cells with 7 neighbors and in light blue, cells with 5 neighbors.

Table 4 detailed data obtained in terms of cone spacing. The space between adjacent cones increased with eccentricity (see Figs 2 and 4). We observed a statistically significant difference (t-test, p<0.001) of 7% between the horizontal and vertical meridians. Cone spacing increased by one third between 2° and 6°.

**Table 4:** Mean ± standard deviation (minimum—maximum) cone spacing, including manual correction, for all eccentricities and all meridians, expressed in μm.

Cone spacing (μm)		2°	3°	4°	5°	6°	Average
**Nasal**		6.281 ± 0.412 (5.430–7.530)	7.108 ± 0.401 (6.210–8.560)	7.753 ± 0.385 (6.880–8.780)	8.175 ± 0.360 (7.290–9.400)	8.476 ± 0.411 (7.570–9.480)	7.560 ± 0.332 (6.746–8.488)
**Temporal**		6.226 ± 0.457 (5.300–7.910)	6.999 ± 0.385 (6.300–8.00)	7.760 ± 0.408 (6.850–8.920)	8.239 ± 0.347 (7.610–9.340)	8.616 ± 0.459 (7.580–9.710)	7.565 ± 0.350 (7.000–8.448)
**Superior**		6.802 ± 0.404 (6.010–7.840)	7.851 ± 0.436 (7.050–9.390)	8.407 ± 0.376 (7.640–9.510)	8.689 ± 0.434 (7.880–9.730)	8.842 ± 0.467 (7.970–10.120)	8.109 ± 0.347 (7.576–9.072)
**Inferior**		6.665 ± 0.412 (5.550–7.940)	7.858 ± 0.414 (6.660–8.920)	8.474 ± 0.376 (7.770–9.540)	8.732 ± 0.376 (7.860–10.000)	8.955 ± 0.420 (7.980–10.130)	8.135 ± 0.342 (7.268–9.042)
**T-test**	**N-T**	p = 0.119	p = 0.003	p = 0.838	p = 0.036	p = 0.001	p = 0.793
	**S-I**	p<0.001	p = 0.938	p = 0.038	p = 0.240	p = 0.011	p = 0.421
	**H-V**	p<0.001	p<0.001	p<0.001	p<0.001	p<0.001	p<0.001

N = nasal; T = temporal; S = superior; I = inferior; H = horizontal and V = vertical.

Based on a Voronoï analysis, the nearest neighbor cones were counted. About 50% of cones had 6 neighbors and 95% had between 5 to 7 neighbors meaning that the cone mosaic array is mainly triangular arranged whatever the eccentricity. Fig 4 shows the Voronoï analysis with the representation of the spatial distribution of cones.

## Discussion

### Automatic versus manual correction

The automatic procedure underestimated the number of cones detected by 6%. This result has been already observed by Garrioch et al. (AOSLO) [30], Talcott et al. (AOSLO) [31], and Jacob et al. (rtx1) [22], who reported a discrepancy of respectively 5.4%, 6% and 8.7%. We observed a larger difference (i.e. up to 10%) close to the fovea which may be due to the higher density of photoreceptors.

### Cone density as a function of eccentricity

**Fig 5:**
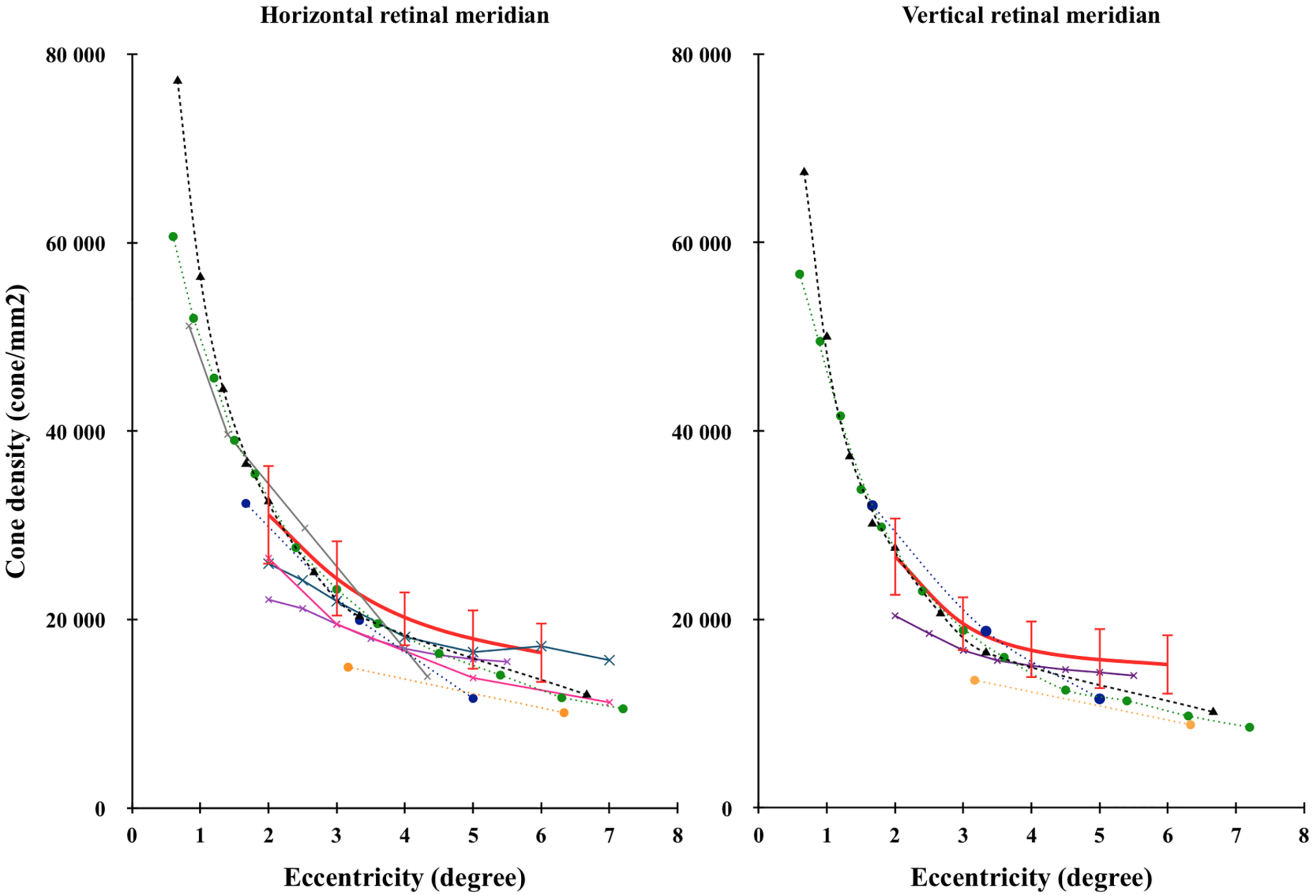
Comparison of cone density as a function of eccentricity with the literature. Horizontal (left) and vertical (right) retinal meridians were compared with the literature. Error bars represent the interval of confidence (± 2SD). The red curve represents our data (rtx1, 109 retinas), the black dashed curve with triangles represents Curcio’s data (histology, 8 retinas) [2], the orange dashed curve with circles represents Chui’s data (AOSLO, 11 retinas) [5], the green dashed curve with circles represents Song’s data (AOSLO, 20 retinas) [7], the blue dashed curve with circles represents Park’s data (AOSLO, 192 retinas) [8], the grey curve with crosses represents Lombardo’s data (rtx1, 20 retinas) [13], the pink curve with crosses represents Muthiah’s data (rtx1, 3 retinas) [17], the purple curve with crosses represents Feng’s data (rtx1, 35 retinas) [20] and the blue curve with crosses represents Jacob’s data (rtx1, 28 retinas) [22].

Fig 5 compared our results (red line) with the literature. Errors bars represent the 95% interval of confidence (± 2 SD). We observed a large variability of cone density among subjects. The inter-individual coefficient of variation (i.e. standard deviation / mean) was in average 11% and 9% respectively in cones/mm^2^ and cones/deg^2^. A large inter-individual variation (i.e. from 12% to 20% in cones/mm^2^) was also observed in the literature [2, 4–8, 13, 14, 16, 32].

The literature exhibits large discrepancy probably due to the large inter individual variability and the low number of subjects involved in each study.

We observed a non linear decrease of cone density with eccentricity in accordance with most of the experiments. On the contrary, Park et al. [8] measured a linear decrease of cone density with eccentricity mainly due to a larger drop at 5° of eccentricity.

### Cone density as a function of meridians

Statistically significant differences were measured at 2°, 3°, 5° and 6° between the nasal and temporal meridians and at 2° and 6° between the superior and inferior meridians. However, these differences were always negligible (i.e. less than 4%). This result is in accordance with other authors, who found a difference ranging from 0.2% to 5.8% in the parafoveal retina (i.e. ∼ 7°) [7, 8, 20].

We observed a difference between both meridians (i.e. higher density along the horizontal meridian) of 14% (4 443 cones/mm^2^), 20% (4 811 cones/mm^2^), 16% (3 227 cones/mm^2^), 11% (2 009 cones/mm^2^) and 8% (1 279 cones/mm^2^) respectively at 2°, 3°, 4°, 5° and 6° (13.8% averaged among the eccentricities). The difference between horizontal and vertical meridians decreased with eccentricity.

The literature exhibits some discrepancies on the difference of cone density between the horizontal and vertical meridians. The majority of authors [2, 5, 7, 15, 20, 21] also measured a difference between both meridians ranging from 9% [2, 21] to 13% [7, 15]. However, it differs from the largest database of Park et al. [8] (AOSLO, from 1.7° to 5°). They observed a smaller difference between both meridians (i.e. 2.3%). It has been previously suggested that this difference could be attributed to the way we use our vision [33–35]. When reading, our horizontal retina is more excited and needs to be more pixelated than our vertical retina. Confirming this hypothesis, other authors [35, 36] measured a higher peripheral visual acuity in the horizontal field than the vertical direction.

We favored another explanation. The differentiation between the horizontal and vertical meridian could originate earlier in evolution. Indeed, da-Costa et al. [37] also reported a higher cone density along the horizontal meridian of Cebus monkey retinas. This higher density should involve a higher performance along the horizontal meridian.

### Cone density as a function of spherical equivalent (SE)

Cone density was not correlated (r^2^ = 0.09 and r^2^ = 0.13 when expressed respectively in visual units and metric units) with refractive error (i.e. spherical equivalent). This can be explained by the poor correlation (r^2^ = 0.40) between the axial length and the refractive error observed in our population. This result is confirmed by the main effects ANOVA analysis (see Table 3).

### Cone density as a function of age

Age doesn’t seem to play an important role on the cone density except at 2° where older persons had a lower cone density by around 7%. The main effects ANOVA analysis confirm this result even if the correlation between cone density and age, at 2° of eccentricity is very poor (r^2^ = 0.10 and r^2^ = 0.07 when expressed respectively in visual units and metric units).

This result is in accordance with Curcio et al., [2, 38] Park et al., [8] and more recently Jacob et al. [22] who did not find significant impact of age. However, at 2° of eccentricity, we slightly differ from these experiments [2, 8, 22, 38] since we observed a statistically significant difference of 7% between the younger and older group. Song et al. [7] also measured a statistically significant difference of 6% in the more central area (i.e. 500 μm of eccentricity ~ 1.7°).

Group 3 (i.e. 41 to 50 years) obtained the smallest densities whatever the eccentricity (see Fig 3). This surprising result was due to the highest average axial length measured on this group. When expressed in cones/deg^2^, this group did not differ from the other groups except at 2° (see Fig 3).

To summarise, age may play a modest role but only in the area closest to the fovea.

### Cone density as a function of axial length

Averaged among each meridian and eccentricity, axial length was highly correlated (r^2^ = 0.60) to cone density when expressed in metric units but poorly (r^2^ = 0.05) when expressed in visual units. When the axial length increases by 1 mm, cone density is reduced by around 1500 cones/mm^2^. The level of correlation is quite similar (i.e. ranging from 0.45 to 0.60) when considering each eccentricity separately. The main effects ANOVA analysis confirms this result (see Table 3).

The literature is not unanimous about the impact of the axial length on cone density in metric units. Indeed, even if all experiments show a negative correlation between cone density (cone/mm^2^) and axial length, the level of correlation (i.e. r^2^) differs remarkably, it ranged from 0.14 [8] or 0.16 [19] to 0.40 [23] or 0.56 [5] at an eccentricity of 2° or 3° and even 0.75 [6] at 1° of eccentricity.

The link between axial length and cone density expressed in metric unit suggests that retina is stretched during the axial elongation of the eyeball. Consequently, the number of cones remains constant while the area covered by the retina increases resulting in a lower number of cones per unit of surface.

From our point of view, cone density should be expressed in visual units instead of metric units to be compared across studies. Indeed, axial length induces an important source of variability between individuals.

### Cone density as a function of gender

We did not observe any difference in cone density, either in metric and visual units. The main effects ANOVA analysis confirms this result.

### Cone spacing

The mean cone spacing increased with eccentricity from 6.5 μm (i.e. 1.35 in minutes of arc) at 2° to 8.7 μm (i.e. 1.81 in minutes of arc) at 6°. This result is in accordance with the literature. Muthiah et al. [17] measured on 3 subjects an average cone spacing ranging from 6.8 μm at 2° to 9.3 μm at 7°. Similarly, Jacob et al. [22] found a mean cone spacing of around 7 μm at 2° and 8.5 μm at 6°. Lombardo et al. [14] also found quite comparable values (i.e. 5.75 μm at 2° and 8.24 μm at 3.7°).

The inter-individual coefficient of variation was 5.5%, ranging between 4.6% to 6.5% as a function of eccentricity.

Cone spacing is directly linked to cone density (r^2^ = 0.90). As a consequence, the impact of age, gender, axial length on cone spacing as well as the difference of cone spacing between meridians were comparable to the ones observed with cone density.

### Packing arrangement of the cone mosaic

Half of the cells had six neighbors and around 45% of all other cells had 5 or 7 neighbors indicating that the main part of the cell arrays was hexagonal. Consequently, we can consider that the mosaic of the retina was mainly triangularly arranged between 2° and 6° of eccentricity. The average coefficient of variation between individuals was around 15%.

We observed a large variability (i.e. between 31% to 68%) in the proportion of cells with 6 neighbors cells between individuals. The proportion of cells with 6 or with 5 to 7 neighbors slightly decreased with eccentricity, respectively from 53% at 2° to 45% at 6° and from 97% at 2° to 93% at 6°.

This result has already been reported. Muthiah et al. [17] and Jacob et al. [22] observed a decrease of the proportion of cells with 6 neighbors from respectively 51% at 2° to 43% at 7° and from approximatively 48% at 2° to 43% at 6°. Lombardo et al. [14] measured a lower percentage of cells with 6 neighbors, ranging from 46% at ~0.8° to 38% at ~3.5°.

The mosaic of the retina became less regular (i.e. a lower proportion of cells with 6 neighbors) as the interval between cones increased (i.e. a lower density), probably due to the increasing proportion of rods.

## Conclusion

This experiment allowed us to answer the questions left unanswered by the literature. Cone density differed markedly between the horizontal and vertical meridians. We also observed that axial length impacted the cone density expressed in unit of area but not in term of visual angle supporting the hypothesis that the retina is stretched with the eyeball elongation. And finally, age does not seem to impact cone density except at 2°.

However, to fully answer this last question, it could be interesting to include older subjects having healthy retinas (i.e. an age group over 60 years old). In this group, the pupil should be dilated since pupil size is often very small in the elderly, leading to a poor quality of the acquired image.

## Supporting information

S1 File Raw data of all subjects. (XLSX)
